# HIV-Negative Cryptococcal Meningoencephalitis Results in a Persistent Frontal-Subcortical Syndrome

**DOI:** 10.1038/s41598-019-54876-7

**Published:** 2019-12-05

**Authors:** Katherine Traino, Joseph Snow, Lillian Ham, Angela Summers, Laura Segalà, Talia Shirazi, Nadia Biassou, Anil Panackal, Seher Anjum, Kieren A. Marr, William C. Kreisl, John E. Bennett, Peter R. Williamson

**Affiliations:** 1 National Institute of Mental Health, National Institutes of Health, Bethesda, MD USA; 2 Radiology and Imaging Sciences, Clinical Center, National Institutes of Health (NIH), Bethesda, MD USA; 3 Laboratory of Clinical Immunology and Microbiology, National Institute of Allergy and Infectious Diseases, National Institutes of Health, Bethesda, MD USA; 4 Johns Hopkins University Department of Medicine, Baltimore, MD USA; 5 Columbia University, New York, NY USA

**Keywords:** Human behaviour, Meningitis

## Abstract

Twenty-seven previously healthy (of 36 consecutive eligible patients), HIV-negative cryptococcal meningoencephalitis (CM) patients underwent comprehensive neuropsychological evaluation during the late post-treatment period (1.3–4 years post diagnosis), assessing attention, language, learning, memory, visuospatial, executive function, information processing, psychomotor functioning, as well as mood symptoms. Seven of eight domains (all except attention) showed increased percentages of CM patients scoring in the less than 16^th^ percentile range compared to standardized normative test averages, adjusted for education level and age. Comparison with a matched archival dataset of mild cognitive impairment/Alzheimer’s disease patients showed that CM patients exhibited relative deficits in psychomotor and executive function with fewer deficits in memory and learning, consistent with a frontal-subcortical syndrome. MRI evaluation at the time of testing demonstrated an association of lower neuropsychological functioning with ventriculomegaly. These studies suggest that CM should be included in the list of treatable causes of dementia in neurological work ups. Future studies are needed to identify diagnostic and treatment regimens that may enhance neurological function after therapy.

## Introduction

Cryptococcal meningoencephalitis (CM) is a highly fatal fungal infection of the central nervous system (CNS), affecting individuals with known immunosuppression such as HIV, solid organ transplantation, or after treatment with corticosteroids as well as some previously healthy individuals^1^. CM causes an estimated 250,000 deaths globally per year, is responsible for 11% of AIDS-related deaths and is currently the most common cause of non-viral meningitis in the U.S.^1–3^. The disease often begins with headache, nausea, and changes in mental status, is confirmed through lumbar puncture, and treated with antifungal medications including Amphotericin B and flucytosine^4^. Secondary hydrocephalus is common in non-HIV populations, which can require treatment with ventriculoperitoneal shunts^5^. Survival rates in populations without a known immunosuppressive condition range from 44% to 79.8% with unclear long-term effects^6,7^.

Mental status changes in the absence of fever in an adult population is a common presentation in non-HIV associated CM, as illustrated by two published case studies^8,9^. In those studies, some HIV-negative individuals were initially misdiagnosed as having Alzheimer’s disease (AD). However, once these patients were properly diagnosed with CM, expedient antifungal treatment resolved acute mental status changes, although one case reported residual deficits in learning^8^. Some recent group studies also suggest residual neuropsychological (NP) deficits in HIV-positive CM patients, testifying to the destructive ability of the fungus, regardless of the host^10,11^.

There is a paucity of literature examining HIV-negative CM patients’ NP functioning after recovery. Previous studies reported significant differences between CM patients and controls on subscales of the Wechsler Adult Intelligence Scales-III (WAIS-III) and the Cognitive Abilities Screening Instrument (CASI)^12,13^. These studies have attempted to relate overall NP functioning or depression to physiological and anatomical changes including microstructural injury, CSF antigen titer^13,14^ and treatment with ventricular-peritoneal shunts^12,14^. However, the studies lacked assessment of specific residual NP deficits that could help to differentiate CM deficits from other neurologic defects within an otherwise normal population. Given that these NP changes may present in a subtle fashion in adults, it is imperative to contrast their NP profiles to that of dementias such as AD because CM is a treatable condition.

The current study thus sought to characterize residual NP deficits in a consecutive cohort of previously healthy HIV-negative CM patients, which represents an understudied subgroup of afflicted patients in the U.S. This population is also less confounded by accompanying co-morbidities typical of HIV infections or underlying conditions requiring immunosuppressive therapy. We then sought to compare the cohort to patients with a well-known cortical dementia to better define the type of residual deficits associated with CM. Using an archival dataset of Pittsburg Compound B-positive (PIB+) AD and mild cognitive impairment (MCI) participants, CM and MCI/AD patients were again matched on severity and compared across NP domains. We also associated NP severity scores with indicators of CM disease severity including cerebrospinal (CSF) glucose, protein, and white blood cell levels; days from symptom presentation to diagnosis; and the axonal damage marker, neurofilament light chain (NFL), measured in CSF as well as neuroimaging findings of gliosis and ventriculomegaly.

## Methods

### Patients

Twenty-seven (19 males, 8 females; ages 22–80, median = 51.6 yr [IQR: 45–62]) previously healthy, HIV-negative CM patients post-acute successful fungal eradication treatment with central nervous system involvement (Tables 1 and 2) consented for additional testing and were referred for NP testing. Patients were diagnosed with CM between January 2010 and August 2016 and were recruited from an ongoing National Institute of Allergy and Infectious Disease study, *Cryptococcosis in Previously Healthy Adults* (NCT00001352). All patients were seen at the National Institutes of Health Clinical Center, Bethesda, Maryland, and informed consent was obtained. All experimental protocols were approved by the research ethics committee and Institutional Review Board of the NIAID (NIH). All methods were carried out in accordance with relevant guidelines and regulations. To be included in the study, patients had to meet the following criteria: (1) have had active *Cryptococcus* infection diagnosed by fungal culture and/or cryptococcal antigen testing without known immune deficiencies, (2) an age of 18 years or older, (3) have a primary physician outside of NIH, (4) agree to genetic testing, and (5) allow sample storage for future research. Additionally, patients included in this study had no history of a CNS infection or head injury earlier in life. Of the 36 patients referred for NP testing, four were excluded from testing for the following reasons: one died of a ruptured left middle cerebral artery; one could not return to the NIH due to pulmonary embolisms that precluded extended travel; one had complete deafness and an inability to see, precluding testing; and one had a mixed AD and CM picture, complicating analysis. From the remaining 32 eligible patients, 27 consented to undergo NP testing (84%). Written informed consent was obtained and was approved by the research ethics committee of the NIAID institutional review board. Initial NP exams were conducted at least 6 months after diagnosis (median = 2 yr [IQR: 1.5–4 yr]).

**Table 1:** Cryptococcal meningoencephalitis versus mild cognitive impairment/Alzheimer’s disease.

	CM patients (*n* = 27)	MCI/AD patients (*n* = 32)	*p*-values*
Age (Range, Med [IQR])	22 to 80, 52.0 [45.0–62.0]	49 to 89, 71.5 [65.5–77.0]	<0.001
Years of Education (Range, Med [IQR])	6 to 16, 12.0 [12.0–13.3]	10 to 20, 16.5 [16.0–20.0]	0.002
Gender (n, % Male)	19, 70.4%	18, 56.3%	0.264
Race/Ethnicity (n, % White)	19, 70.4%	23, 71.9%	0.442
Overall Average T-score (Mean ± SD)	43.1 ± 8.2	41.5 ± 5.6	0.427
Beck Depression Inventory (Med [IQR])	11.0 [6.0–20.0]	5.0 [0.8–7.3]	0.001
Beck Anxiety Inventory (Med [IQR])	12.5 [6.3–19.3]	2.0 [0.8–4.3]	<0.001
Wechsler Test of Adult Reading (Med [IQR])	98.5 [89.0–114.0]	116.5 [104.0–122.0]	0.014

BDI within normal limits ≤13; BAI within normal limits ≤7. *p-values are based on independent samples *t*-tests for continuous variables and *χ*^2^ analyses for categorical variables.

**Table 2:** Cryptococcal meningoencephalitis patients (*N* = 27). Med = median, IQR = interquartile range, WNL = within normal limits, NP = neuropsychological.

Patients with *C. gattii* (n, %)	4, 14.8%
Time from diagnosis to NP testing, years (Med [IQR])	2.0 [1.5–4.0]
Patients with fever (n, %)	5, 18.5%
Change in mental status (n, %)	7, 25.9%
Headache (n, %)	20, 74.1%
Malaise (n, %)	21, 77.8%
CSF Glucose nadir (Med [IQR])	37.0 [24.0–48.5]
*CD4 (Med [IQR])	532 [291–927]
*CD8 (Med [IQR])	337 [190–600]
Blood cryptococcal antigen titer (Med [IQR])	1:64 [1:2–1:256]
CSF cryptococcal antigen titer (Med [IQR])	1:64 [1:5–1:320]
Patients with ≥4 weeks of Amphotericin B (n, %)	22, 81.5%
Patients with Shunts (n, %)	9, 33.3%
Patients with Contrast Enhancement (n, %)	12, 44.4%
Patients with Frontal Enhancement (n, %)	1, 3.7%
Patients with Basal Ganglia Enhancement (n, %)	4, 14.8%
Patients with Cerebellar Enhancement (n, %)	2, 7.4%
Patients with Intra Axial Enhancement (n, %	7, 25.9%
Patients with Frontal Volume Loss (n, %)	3, 11.1%
Patients with Cerebellar Volume Loss (n, %)	3, 11.1%
Patients with Overall Volume Loss (n, %)	11, 40.7%
Patients with Basal Ganglia Hemorrhage (n, %)	2, 7.4%
Patients with Ventriculomegaly (n, %)	16, 59.3%
Patients with Non-Enhancing Gliosis (n, %)	20, 74.1%

*Labs collected upon patient presentation to NIH.

The archival dataset of C-Pittsburgh Compound B (PIB) positive MCI and AD patients (Table 1) originally included 58 participants from a PET imaging/dementia study (NCT00955422), a subset of which was later used to create a matched sample of cortical dementia patients for comparison to the CM group. PET imaging using PIB was used to quantify beta-amyloid plaques, which were used in identifying participants as PIB+ according to an adaptation from Jack and colleagues (2008)^15,16^. Performance validity, as measured by the Medical Symptom Validity Test, was assessed in 85% of CM and 78% of MCI/AD cases. Most patients in both groups passed validity testing outright, scoring above cutoff scores. In both groups, a few patients were classified as having a (1) severe impairment profile or (2) uncertain validity profile of performance, however, since the performance in these cases was clinically consistent with their NP impairment, no one was excluded based on performance validity concerns.

### Procedures

#### Assessments

A trained psychometrist, supervised by a board-certified clinical neuropsychologist (JS), administered a comprehensive NP evaluation to each (CM as well as MCI/AD) patient. The administered tests (for both the CM and MCI/AD patients) were grouped into domains on a theoretical basis. The domains included: psychomotor, information processing, executive function, learning, memory, language, attention, visuospatial, and mood symptoms (see Table 3 for specific NP measures grouped by domain). Tests were scored and quality-assured by a trained psychometrist and research assistant. The Revised Comprehensive Norms for an Expanded Halstead-Reitan Battery (Version 4.01)^17^, Calibrated Neuropsychological Normative System (Version 1.10)^18^, and WAIS III WMS III Writer (Version 1.0)^19^ scoring software programs, as well as testing manual norms, were used to calculate demographically-corrected scores for age, sex, race/ethnicity and education. These corrections are based on published and commercially available norms derived from generally large and representative samples widely used in neuropsychology. Overall domain scores were determined by averaging across measures within each domain.

**Table 3:** Neuropsychological evaluation battery.

Neuropsychological domain	Neuropsychological test	Normative data
Psychomotor	Grooved Pegboard	EHRB
Information Processing	Trail Making Part A Symbol Digit Modalities Test	EHRB SDMT Western Psychological Services Norms
Executive Function	Trail Making Part B Wisconsin Card Sorting Test – Perseverative Responses	EHRB WCST Software Norms
Learning	Hopkins Verbal Learning Test – Revised, Total Recall Brief Visual Memory Test – Revised, Total Recall	CNNS CNNS
Memory	Hopkins Verbal Learning Test – Revised, Delayed Recall Brief Visual Memory Test – Revised, Delayed Recall	CNNS CNNS
Language	Controlled Oral Word Association Test Boston Naming Test	EHRB EHRB
Attention	Wechsler Memory Scale III – Digit Span	WAIS-III/WMS-III Writer Software Norms (Version 1.0)
Visuospatial	Wechsler Abbreviated Scale of Intelligence – Original/II Block Design	WASI Original/II Scoring Manual Norms
Average T-Score	Average of all above-listed scores	*Not applicable*
Premorbid Intellectual Functioning	Wechsler Test of Adult Reading, Standard Score and Demographics-Predicted Score	WTAR Scoring Manual Norms
Performance Validity	Medical Symptom Validity Test	*Not applicable*
Mood Symptoms	Beck Depression Inventory – II, Beck Anxiety Inventory	*Not applicable*

EHRB = Expanded Halstead-Reitan Battery Software Norms (Version 4.01), CNNS = Calibrated Neuropsychological Normative System Software Norms (Version 1.10).

Each patient also completed a physical examination, medical history, routine blood tests, and lumbar puncture for CSF. A clinical magnetic resonance imaging (MRI) scan was collected for each patient. A board-certified diagnostic neuroradiologist’s judgment, blinded to the results of laboratory biomarkers was used to determine presence/absence of shunts, leptomeningeal enhancement, intraparenchymal enhancement and its location, presence of cerebral and or cerebellar volume loss, presence of hemorrhage and its location if present, the presence of non-enhancing gliosis, and the presence of obstructive hydrocephalus or ventriculomegaly in the presence of cerebral atrophy. Multiplanar, multisequence images of the brain were reviewed using sagittal T1, axial T1 pre and postcontrast, axial T2, axial FLAIR postcontrast, sagittal T1 3D postcontrast weighted images of the brain using a 3 Tesla MRI. CSF levels of NFL were measured according to the manufacturer’s instructions using a sandwich ELISA method (Uman Diagnostics AB; Umea Sweden)^20^.

#### Statistical analyses

Demographically-corrected T-scores (*M* = 50, *SD* = 10) from the NP evaluation were averaged from the respective tests to calculate NP domain T-scores as well as an overall average T-score (Table 3). Scores that are 1 SD below the mean (≤16^th^ percentile) suggest possible impairment; scores that are 2 SD below the mean (≤2^nd^ percentile) are highly suggestive of impairment.

Statistical analyses were conducted using IBM SPSS Statistics (version 24). First, analyses were conducted to explore relationships between biological measures and NP data. We used Pearson correlations to investigate relationships between NP test data and CSF glucose, CSF protein, CSF white blood cell count, and length of time from symptom presentation to diagnosis and treatment. Independent samples *t*-tests were conducted to compare NP test data between groups based on the presence of shunts, MRI enhancements (overall, basal ganglia, cerebellar, and intra axial), overall brain volume loss, basal ganglia hemorrhage, obstructive hydrocephalus, non-enhancing gliosis, and ventriculomegaly.

For comparison to AD, we utilized an archival dataset of MCI/AD patients who were administered the same NP battery as the CM patients. We used the overall average T-score for individuals to choose a subsample of the MCI/AD patients who were similar to the CM patients in terms of their average level of functioning or overall impairment. Originally, the entire MCI/AD sample was compared to the CM sample based on overall average T-score; the average T-score for the CM sample was 43.1 ± 8.2 and 34.7 ± 9.3 for the original MCI/AD archival dataset sample. We then selected MCI/AD patients with an equivalent overall average T-score to that of the CM patients’ overall average T-score. As a result, we removed 26 subjects with low scores from the MCI/AD sample to match the average T-score to that of the CM sample. After doing so, the MCI/AD sample contained 32 PIB+ MCI/AD patients with an overall average T-score of 41.5 ± 5.6, matched to the CM patients based on overall average T-score (Table 1). Using an identical NP testing battery, demographically-corrected T-scores were calculated for each NP domain (Table 3) in both the MCI/AD sample and the CM sample. Analyses of covariance, controlling for significant group differences in depression and anxiety scores, were conducted to compare MCI/AD patients with CM patients on NP domain T-scores. Due to individual T-scores being demographically adjusted for sex, age, race/ethnicity, and education, we did not control for significant group differences in age, education, or WTAR scores in analyzing comparisons.

## Results

### Study subjects

Table 2 shows that the 27 patients had normal CD4+ and CD8+ lymphocyte counts (532 cells/μl[IQR: 291–927]; 337 cell/ml [IQR: 190–600]) and two had antibodies to GMCSF as reported previously^21^. Patients presented for diagnosis initially often with headache (74.1%) and malaise (77.8%), although only 5 patients had a fever (18.5%). Altered mental status was also uncommon during the initial presentation prior to transfer to the NIH, exhibited in only 7 (25.9%) patients, contrary to what has been reported in the literature^8,9^. Laboratory findings during the initial treatment period demonstrated abnormally low CSF glucose (37 mg/dL [IQR: 24.0–48.5], Table 2) as well as diagnostic CSF cryptococcal antigen tests, typical for CM. Patients were uniformly treated with at least 4 weeks of amphotericin B preparations with fluconazole oral therapy for at least 1 year. All patients had negative CSF cultures at the time of NP and MRI exams.

#### Neuropsychological testing demonstrates profound defects in neurological functioning in CM

**Figure 1:**
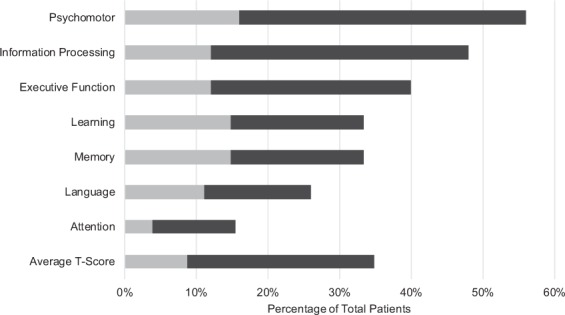
Percentage of impaired cryptococcal meningoencephalitis (CM) patients in each neuropsychological (NP) domain. CM patients were scored either less than the 16th percentile (darker grey bar) and within that, less than the 2nd percentile (lighter grey bar). Comprehensive NP testing of CM patients identifies deficits in the less than 16th percentile range in all domains except attention (15.4%). Results of NP functioning were classified by domain in 27 CM patients and given a domain score by averaging T-scores for the NP tests within the respective NP domain.

CM patients had, on average, a high school level education as well as average estimated premorbid IQ (WTAR standard score = 100.7 ± 14.7, medians are reported in Table 1). After calculating the average T-score for each domain, we determined the percent of CM patients who scored one standard deviation below the mean (i.e., the 16^th^ percentile) or more. As shown in Figure 1, all domains except attention (15.4%) display increased percentages of CM patients scoring in the less than 16^th^ percentile range (25.9% in language to 56.0% in psychomotor). Therefore, the data suggest that the CM patients, on average, demonstrated higher levels of impairment on NP testing than expected relative to standardized normal population test averages. Similarly, we found a low incidence (3.8% in attention to 16.0% in psychomotor) of CM patients who scored below two standard deviations below the mean (i.e., the 2^nd^ percentile) for each domain in comparison to what would be expected in the general population.

#### Neuropsychological comparison with MCI/AD patients suggestive of a frontal-subcortical syndrome in CM

**Figure 2:**
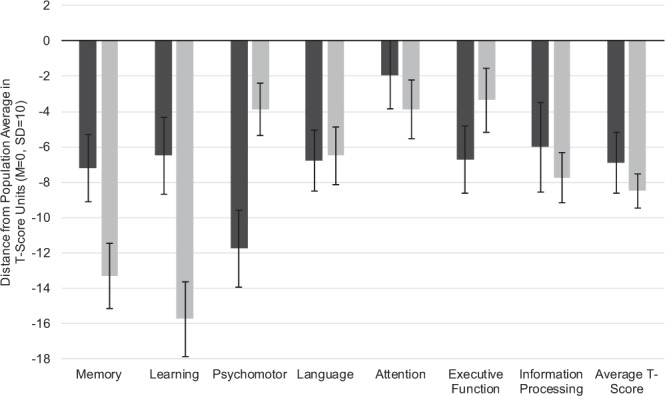
Neuropsychological (NP) composite scores for cryptococcal meningoencephalitis (CM; darker grey bar) survivors compared with Pittsburgh Compound B-Positive (PIB+) mild cognitive impairment/Alzheimer’s disease (MCI/AD; lighter grey bar) patients. NP scores of a cohort of previously healthy, HIV-negative patients with cryptococcal meningoencephalitis (*N* = 27) were compared to a sample of PIB+ MCI/AD patients (*N* = 32), matched on average T-score. NP comparison with MCI/AD patients suggests relative deficits in psychomotor and executive function domains in CM patients. Significant differences between CM and MCI/AD patients were identified for memory, *F*(1,50) = 8.50, *p* = 0.005, and learning domains, *F*(1,50) = 11.47, *p* = 0.001, after controlling for mood symptoms.

Next, we compared our CM patients to our (T-score matched) archival dataset of MCI/AD patients. There were significant differences between groups on self-report measures of depression, *t*(40) = 3.56, *p* = 0.001, and anxiety, *t*(33) = 5.36, *p* < 0.001, with CM patients reporting 6 and 10.5 more symptoms, respectively, out of 63 possible (Table 1). Therefore, we controlled for both depression and anxiety in analyses of covariance (ANCOVA). One-way ANCOVAs were conducted to determine statistically significant differences between CM and MCI/AD groups on NP domains controlling for depression and anxiety (Table 4). After controlling for depression and anxiety, there was a significant effect of group status on memory, *F*(1,50) = 8.50, *p* = 0.005 and learning, *F*(1,50) = 11.47, *p* = 0.001. MCI/AD patients scored lower on both memory and learning domains compared to CM patients (Fig. 2). Both memory (*η*_*p*_^2^ = 0.15) and learning (*η*_*p*_^2^ = 0.19) domains demonstrated medium effects. CM patients performed significantly worse than MCI/AD patients in psychomotor functioning (*p* = 0.003), but this effect became non-significant after accounting for depression and anxiety. There was also a trend towards a significant difference in attention between groups (*p* = 0.083), such that patients with MCI/AD had worse attention. Figure 2 demonstrates CM and MCI/AD group averages from the normative population mean for each domain. CM patients exhibited relative deficits in psychomotor and executive function domains, with executive function being the most affected domain, consistent with a frontal-subcortical syndrome. On the other hand, and as expected, the MCI/AD group demonstrated hallmark deficits of a cortical dementia syndrome, with memory and learning being the most affected domains.

**Table 4:** One-way analysis of covariance results for differences between CM and MCI/AD groups on NP domains controlling for depression and anxiety.

Predictor	Sum of Squares	*df*	Mean Square	*F*	*p*-value	partial η^2^
***Memory***						
(Intercept)	33633.48	1	33633.48	360.05	<0.001	0.878
Patient group	794.14	1	794.14	8.50	0.005	0.145
Error	4670.71	50	93.41			
***Learning***						
(Intercept)	34644.81	1	34644.81	270.97	<0.001	0.844
Patient group	1466.13	1	1466.13	11.47	0.001	0.187
Error	6392.65	50	127.85			

Only memory and learning domains exhibited significant differences between groups with MCI/AD patients demonstrating worse performance in the domains compared to CM patients.

#### Associations of lower neuropsychological scores and residual findings on Magnetic Resonance Imaging

CM patients had numerous brain MRI findings. As indicated in Table 2, 44.4% had contrast enhancement, 59.3% had ventriculomegaly with 40.7% attributable to readily apparent volume loss, and 74.1% had non-enhancing gliosis. See Table 2 for further details.

**Figure 3:**
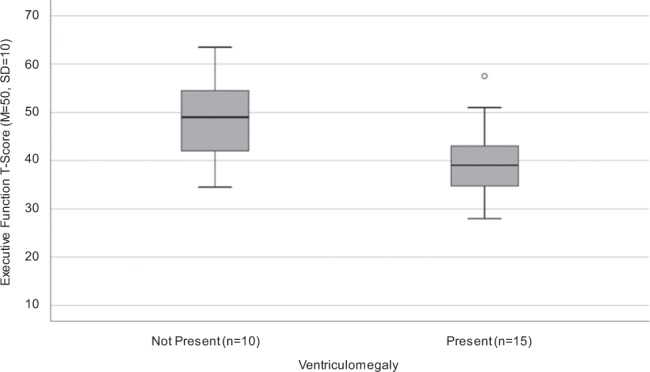
Executive function T-scores vary with the presence of ventriculomegaly in cryptococcal meningoencephalitis (CM) patients. Magnetic resonance imagining was utilized to assess ventriculomegaly (i.e. enlarged ventricles). As evaluated by neuropsychological examination, CM patients with ventriculomegaly scored significantly lower on executive function, *t*(23) = 2.59, *p* = 0.017. Of note, two patients did not complete both tests of executive function and therefore were not included in this comparison (one patient had ventriculomegaly and the other did not).

Contrasting CM patients across MRI measures – ventriculomegaly, brain volume loss (overall, frontal, and cerebellar), basal ganglia hemorrhage, presence of obstructive hydrocephalus and non-enhancing gliosis – one significant between-groups difference by univariant analysis on NP evaluation was found. In particular, there was a statistically significant difference between those with and without ventriculomegaly present on MRI on executive functioning, *t*(23) = 2.59, *p* = 0.017, with patients with ventriculomegaly scoring one standard deviation lower on executive functioning on average (Fig. 3). Ventriculomegaly was felt to be due predominantly to brain volume loss as all CM patients had normal intracranial pressures at the time of NP testing. The effect size for this analysis (*d* = 1.05) was found to exceed Cohen’s (1988)^22^ convention for a large effect (*d* = 0.80). Of note, a difference between patients with and without non-enhancing gliosis trended towards significance for psychomotor function, *t*(23) = 2.04, *p* = 0.053, with those with non-enhancing gliosis performing nearly one standard deviation lower. On secondary analysis, CM patients with ventriculomegaly were older in age than those without ventriculomegaly (median: 58.0 years vs 45.0 years), *t*(25) = 4.17, *p* < 0.001. Overall average T-score did not correlate with age, *r* = 0.08, *p* = 0.709, which is expected for a T-score adjusted for age. Overall average T-score also did not differ on the basis of ventriculoperitoneal shunting, *t*(13) = 1.26, *p* = 0.230.

#### Biomedical Illness Indicators

Only 11 of the 27 CM patients had CSF NFL levels for analysis. Despite being a small sample, there was a strong negative correlation between NFL and age (*r* = −0.89, *p* < 0.001). There were no significant Pearson correlations between NP tests and clinical indicators of illness severity, except for a positive correlation between attention and length of time from symptom presentation to diagnosis and treatment (*r* = 0.65, *p* = 0.023). However, this result was found with a smaller subsample of CM patients (*n* = 12) among many other non-significant correlations that were conducted between NP tests/domains and biomedical illness indicators, such as CSF glucose, CSF protein, and CSF white blood cell count (*p* > 0.05). Thus, it is difficult to draw any firm conclusions from this single, significant correlation.

## Discussion

The present study sought to characterize NP functioning in 27 HIV-negative previously healthy CM patients and perform a discovery study to correlate NP findings to biomedical and neuroimaging measures. The study represents the largest detailed NP study to date of this patient population. We found that, despite successful fungal eradication with standard-of-care regimens, HIV-negative CM patients demonstrated higher overall rates of NP impairment compared to standardized population norms. Limiting the study to a previously healthy patient population strongly suggests that these deficits are due to the acquired infection rather to underlying co-morbidities common in transplant populations and HIV. These results were present during the multi-year observational period, well beyond that of the initial 4-week antifungal therapy. Furthermore, detailed study showed that CM patients’ NP functioning profile demonstrated significant differences in memory and learning compared to that of an average T-score-matched group with MCI/AD. CM patients’ performance also suggested a frontal-subcortical syndrome with impairment of psychomotor skills, information processing, and executive functions according to established neurological categories^23^. Upon relating NP variables to various biomedical and neuroimaging measures, significant between-groups differences were found on univariant testing for ventriculomegaly on executive functioning. However, due to the discovery nature of the study and small sample size, adjustments for multiple comparisons were not able to demonstrate statistically significant differences. Ventriculomegaly in the absence of elevated central pressures has been implicated in chronic neurological dysfunction previously and these data extend this finding to deficits in CM^21^.

Previous studies reported significant differences between CM patients and controls on subscales of the WAIS-III and the CASI^12,13^. In contrast, the current study implemented a comprehensive NP testing battery by using domain-specific tests to better detect performance rather than general intellectual abilities. In addition, although patient studies are methodologically strongest when they employ a carefully demographically-matched control group, when that is not possible, an acceptable methodological second option (when determining NP impairment) is to employ robust demographically-corrected normative values. Thus, the current study compared CM patients’ performance to that of demographically-corrected (age, gender, race/ethnicity, and education) population norms. M.-H. Chen *et al*.^12^ and Lu *et al*.^13^ found differences in NP performance between CM patients and an age- and sex-matched control group. However, it is unclear whether these group differences are due to CM or if they are a result of premorbid differences. Though non-significant, differences in education between groups could support that premorbid differences in cognitive functioning were reflected in their NP performance^12,13^. Tests of crystallized intelligence—such as WAIS-III Information, Vocabulary, and Comprehension subtests—are usually employed as measures of premorbid functioning as global degenerative processes do not typically affect them. In M.-H. Chen *et al*.^12^ and Lu *et al*.^13^, controls demonstrated better performance on tests of fluid, but also crystallized abilities which could have suggested that better performance in controls was due to premorbid superiority and not entirely secondary to CM.

The current study compared CM patients’ NP performance profile to that of a well-known cortical dementia sample of MCI/AD patients. We found significant differences between CM patients and MCI/AD patients with the most prominent effects on memory, learning, and psychomotor domains. Specifically, CM patients performed worse than MCI/AD patients on psychomotor functioning, but this effect became non-significant after accounting for mood symptoms. As expected, MCI/AD patients performed significantly worse than CM patients on memory and learning. The attention domain also trended towards differences between these groups. This comparison is clinically important given case studies^8,9^ reporting that some patients without a known immunosuppressive condition who contract *Cryptococcus* infection have been initially misdiagnosed with AD. As such, defining critical NP differences between these groups may better enable clinicians to provide expedient diagnostic workup. Indeed, a simple blood or CSF cryptococcal antigen test can lead to a diagnosis of this highly treatable condition and thus, CM should be included in the list of potentially treatable dementias. For example, in the present study, 22/27 (81.5%) and 23/27 (85.2%) of patients had positive blood and CSF cryptococcal antigen tests, respectively. This finding is especially important as only 5/27 (18.5%) of this previously healthy patient population presented with fevers that would normally lead to prompt initiation of an infectious workup. In addition, these findings will better enable clinicians to prepare patients for possible residual NP difficulties even after successful antifungal treatment.

As with previous studies, we attempted to relate NP outcomes to CM disease-related findings from blood, CSF and imaging. In doing so, we found significantly worse performance on executive function in CM patients with ventriculomegaly compared to those without. Previous CM studies have also attempted to relate NP assessment results to disease-related biomedical and imaging findings. Lu *et al*.^13^ reported an indirect relationship between NP performance and CSF antigen titer. Initial cryptococcal antibodies were associated with worse microstructural injury in the brain on diffusion tensor imaging (DTI), and DTI was related to poorer follow-up NP performance. C.-H. Chen *et al*.^14^ found an inverse correlation between WAIS-III block design and initial cryptococcal antigens with higher levels correlating to worse block design performance. Similarly, prior studies have examined the role of shunting and depression on NP scores in CM patients^12,14^. M.-H. Chen *et al*.^12^ observed significant differences between CM patients with and without shunts on WAIS-III and CASI subtests, such that patients with shunts demonstrated worse NP performance on most (but not all) of these subtests. C.-H. Chen *et al*.^14^ reported that CM patients with depression performed worse than CM patients without depression on executive function and visuoconstruction domains, in addition to having a higher rate of CM-related lesions on MRI. The present study examined CSF results, shunt versus no shunt, and depression versus no depression in CM patients. We were not able to replicate findings reported by previous groups except for DTI, which was lacking in present study. These disparate results could be due to differences in treatment regimens including the timing of shunting after diagnosis of elevated central pressures or the use of corticosteroids^12^, both of which were instituted early in our cohort. Differences could also be explained by differences in NP testing batteries, as reported earlier, and differing disease states at patient presentation (acute versus late).

The current study has several limitations. Only a portion of the larger HIV-negative CM patient cohort (27 of 36) underwent NP evaluation due to lack of consent to the 3-hour evaluation by five patients. Four additional patients were not eligible as one died during the acute illness, one could not return due to travel-related pulmonary embolisms, one had complete deafness and thus was unable to perform testing and one had a documented mixed AD which would complicate testing analysis, leaving 32 eligible patients. The CM patients’ data in this study were collected after the acute infection phase and there were no data available for the patients’ pre-infection NP status, other than a measure of estimated premorbid intellectual functioning collected during the NP assessment. Additionally, long-term follow-up data were not collected for each patient to track NP symptom persistence although the evaluation late after disease onset suggests significant persistence. While the sample size is larger than previously reported CM studies, it was not sufficient to conduct both a discovery study and confirm the observed differences with multi-variate testing. The neuroimaging data collected were also from a clinical MRI scan rather than using functional imaging technology, and thus cannot offer insight into CSF flow-and-brain interaction nor allow for directly, anatomically correlating brain lesions to NP domains. We attempted to look at intra axial enhancement in particular lobes (e.g., frontal, temporal, parietal, and occipital) by defining whether enhancement was present in each lobe for each subject. However, the small sample sizes made it impossible to perform statistical analyses: only two subjects had enhancement in the frontal lobes, one in the temporal lobes, three in the parietal lobes, and one in the occipital lobes. Even fewer subjects had hemorrhage in those respective lobes. This study also did not include a sample of matched healthy controls for comparison; however, demographically-adjusted normative population data were used to analyze NP testing performance. Finally, when comparing the CM patients’ frontal-subcortical syndrome presentation, an MCI/AD cortical dementia sample was used rather than a sample of patients with known frontal-subcortical syndromes. We chose this comparison sample simply due to archival data availability. Nevertheless, though the sample size is small, it is larger than similar samples currently in the literature, and utilizes a comprehensive NP exam coupled with the numerous biomedical and neuroimaging variables available, thus adding to the existing body of literature on NP functioning and CM. Future studies should examine patient biological markers throughout infection status, and include long-term NP follow-up, enhanced imaging technology, a matched healthy comparison group, and a larger sample size.

In conclusion, our findings demonstrate that HIV-negative CM patients exhibit significant NP functioning impairment suggestive of a frontal-subcortical syndrome. Among patients, ventriculomegaly appeared to be a potential surrogate for deficits in executive function which will require validation. Additional correlations such as between gliosis on MRI, CSF surrogate biomarkers such as NFL, and psychomotor functioning will require further study.

## References

[1] Pyrgos, V., Seitz, A. E., Steiner, C. A., Prevots, D. R. & Williamson, P. R. Epidemiology of Cryptococcal Meningitis in the US: 1997–2009. *PLoS ONE* **8** (2013). 10.1371/journal.pone.0056269 PMC3574138 23457543

[2] Castelblanco RL, Lee M, Hasbun R (2014). Epidemiology of bacterial meningitis in the USA from 1997 to 2010: a population-based observational study. The Lancet Infectious Diseases.

[3] Rajasingham R (2017). Global burden of disease of HIV-associated cryptococcal meningitis: an updated analysis. The Lancet Infectious Diseases.

[4] Williamson PR (2016). Cryptococcal meningitis: epidemiology, immunology, diagnosis and therapy. Nature Reviews Neurology.

[5] Mehta GU (2017). Corticosteroids for shunted previously healthy patients with non-HIV cryptococcal meningoencephalitis. Journal of Neurology, Neurosurgery & Psychiatry.

[6] Ecevit IZ, Clancy CJ, Schmalfuss IM, Nguyen MH (2006). The Poor Prognosis of Central Nervous System Cryptococcosis among Nonimmunosuppressed Patients: A Call for Better Disease Recognition and Evaluation of Adjuncts to Antifungal Therapy. Clinical Infectious Diseases.

[7] Shih CC, Chen YC, Chang SC, Luh KT, Hsieh WC (2000). Cryptococcal meningitis in non-HIV-infected patients. QJM: An International Journal of Medicine.

[8] Ala TA, Doss RC, Sullivan CJ (2004). Reversible dementia: A case of cryptococcal meningitis masquerading as Alzheimers disease. Journal of Alzheimers Disease.

[9] Hoffmann M, Muniz J, Carroll E, Villasante JD (2009). Cryptococcal Meningitis Misdiagnosed as Alzheimers Disease: Complete Neurological and Cognitive Recovery with Treatment. Journal of Alzheimers Disease.

[10] Carlson RD (2014). Predictors of neurocognitive outcomes on antiretroviral therapy after cryptococcal meningitis: a prospective cohort study. Metabolic Brain Disease.

[11] Levine AJ (2008). An exploratory study of long-term neurocognitive outcomes following recovery from opportunistic brain infections in HIV adults. Journal of Clinical and Experimental Neuropsychology.

[12] Chen M-H (2015). Long-Term Neuropsychological Sequelae in HIV-Seronegative Cryptococcal Meningoencephalitis Patients with and without Ventriculoperitoneal Shunts: A Cine MRI Study. Behavioural Neurology.

[13] Lu C-H (2011). Assessing the Chronic Neuropsychologic Sequelae of Human Immunodeficiency Virus–Negative Cryptococcal Meningitis by Using Diffusion Tensor Imaging. American Journal of Neuroradiology.

[14] Chen C-H (2012). Neuro-psychological Sequelae in HIV-negative Cryptococcal Meningitis after Complete Antifungal Treatment. Acta Neurologica Taiwan.

[15] Jack CR (2008). C PiB and structural MRI provide complementary information in imaging of Alzheimer’s disease and amnestic mild cognitive impairment. Brain.

[16] Kreisl WC (2013). *In vivo* radioligand binding to translocator protein correlates with severity of Alzheimer’s disease. Brain.

[17] Heaton, R. K., Miller, S. W., Taylor, M. J. & Grant, I. *Revised comprehensive norms for an expanded Halstead–Reitan battery*. (Psychological Assessment Resources, 2004).

[18] Schretlen, D. J., Testa, M. S. & Pearlson, G. D. *Calibrated Neuropsychological Normative System*. (Psychological Assessment Resources, Inc., 2010).

[19] *WAIS-III, WMS-III, WIAT-II WRITER* (Psychological Corporation, 2002).

[20] Panackal AA (2015). Paradoxical immune responses in non-HIV Cryptococcal meningitis. PLoS Pathogens.

[21] Kosa P (2015). Novel composite MRI scale correlates highly with disability in multiple sclerosis patients. Multiple Sclerosis and Related Disorders.

[22] Cohen, J. *Statistical Power Analysis for the Behavioral Sciences*. (Routledge Academic, 1988).

[23] Bonelli RM, Cummings JL (2007). Frontal-subcortical circuitry and behavior. Dialogues in Clinical Neuroscience.

